# Clinical Predictors and Recovery Patterns of Visual Impairment as a Post-Stroke Complication: A Retrospective Single-Center Cohort Study from a Romanian Comprehensive Stroke Unit

**DOI:** 10.3390/jcm15135291

**Published:** 2026-07-07

**Authors:** Mirela Loredana Grigoraș, Sorin Lucian Bolintineanu, Livia Stanga, Laura Andreea Ghenciu

**Affiliations:** 1 Department of Functional Sciences, Victor Babeș University of Medicine and Pharmacy, 300041 Timisoara, Romania; grigoras.mirela@umft.ro; 2 Department of Anatomy and Embryology, Victor Babeș University of Medicine and Pharmacy, Eftimie Murgu Square No. 2, 300041 Timisoara, Romania; s.bolintineanu@umft.ro; 3 Department III, Discipline of Pathophysiology, Faculty of Medicine, Victor Babeș University of Medicine and Pharmacy, 300041 Timisoara, Romania; 4 Department of Infectious Diseases, Victor Babeș University of Medicine and Pharmacy, 300041 Timisoara, Romania; bolintineanu.laura@umft.ro; 5 Center for Translational Research and Systems Medicine, Victor Babeș University of Medicine and Pharmacy, 300041 Timisoara, Romania

**Keywords:** stroke, vision disorders, hemianopsia, recovery of function, prognosis

## Abstract

**Background/Objectives:** Visual impairment is an underrecognized but functionally disabling complication of stroke that adversely affects rehabilitation potential, autonomy, and quality of life. Clinical, anatomical, and ophthalmologic determinants of post-stroke visual recovery remain incompletely defined, particularly in Eastern European tertiary stroke units where structured visual follow-up is not standardized. This study aimed to identify clinical, imaging, and ophthalmologic predictors of favorable visual recovery and to evaluate whether integrating these domains improves early prognostic stratification beyond standard neurological assessment. **Methods:** We conducted a retrospective single-center cohort study of 71 consecutive adult patients admitted with acute stroke and a documented visual complication between January 2022 and September 2025 at Pius Brinzeu Emergency County Hospital and Victor Babes University of Medicine and Pharmacy Timisoara. Favorable recovery was defined as ≥50% improvement in visual field index (VFI) at 6 months. Group comparisons used Student’s *t*-test, Mann–Whitney U test, chi-square test, and Fisher’s exact test. Multivariable logistic regression, Cox proportional hazards modeling, and unsupervised k-means clustering were performed. **Results:** Twenty-nine patients (40.8%) achieved favorable recovery, while 42 (59.2%) had persistent impairment. Responders were younger (62.8 ± 10.7 vs. 70.4 ± 10.8 years, *p* = 0.005) and had lower admission National Institutes of Health Stroke Scale (NIHSS) (6.4 ± 2.9 vs. 10.3 ± 4.4, *p* < 0.001), smaller lesion volumes (18.7 ± 11.4 vs. 33.2 ± 18.7 mL, *p* < 0.001), thicker peripapillary retinal nerve fiber layer (89.3 ± 7.6 vs. 78.2 ± 9.4 μm, *p* < 0.001), and earlier rehabilitation initiation (11.4 ± 5.3 vs. 21.7 ± 9.8 days, *p* < 0.001). NIHSS, time to rehabilitation, and optical coherence tomography (OCT) pRNFL thickness remained independent predictors. The full integrated model achieved an area under the receiver operating characteristic curve (AUC) of 0.87. Cluster analysis identified three distinct phenotypes with favorable recovery rates of 79.2%, 34.8%, and 8.3%. **Conclusions:** Combined clinical, neuroimaging, and ophthalmologic profiling—particularly OCT pRNFL—meaningfully refines early prediction of post-stroke visual recovery and supports phenotype-driven rehabilitation planning.

## 1. Introduction

Stroke remains a leading cause of long-term disability worldwide, and the burden of stroke-related complications increasingly drives both individual prognosis and healthcare system costs [1,2]. Among these complications, post-stroke visual impairment is often clinically under-detected, despite affecting an estimated 20–60% of stroke survivors when systematically assessed [3,4]. Visual deficits range from homonymous hemianopia and quadrantanopia to cortical visual dysfunction, oculomotor palsies, and complex disorders of higher-order visual processing such as visual neglect and visual agnosia. These impairments compromise reading, mobility, driving capacity, fall risk, return to work, and overall quality of life, and they substantially limit participation in conventional rehabilitation programs that themselves rely on intact visual function.

Despite this functional importance, post-stroke visual care remains uneven across centers, with limited integration between stroke neurology, neuro-ophthalmology, and visual rehabilitation services [5,6]. In many comprehensive stroke units, visual deficits are documented bedside through confrontational testing or simple acuity measurement, but quantitative visual field assessment, structural ophthalmologic imaging, and standardized vision-related quality of life measurement remain infrequent. As a result, clinicians often face the parallel difficulty of identifying which patients are likely to recover spontaneously versus those requiring early intensive rehabilitation, prism therapy, compensatory training, or visual restitution interventions. Improving early prognostication is therefore a key step toward more individualized post-stroke visual care.

The biological rationale for early prediction is supported by accumulating evidence that microstructural integrity of the visual pathway influences post-stroke recovery potential. Optical coherence tomography (OCT) studies have demonstrated that retrograde trans-synaptic degeneration of retinal ganglion cells follows damage to retrochiasmal visual pathways, with measurable thinning of the peripapillary retinal nerve fiber layer (pRNFL) and ganglion cell layer (GCL) within weeks of cortical or optic radiation injury [7,8]. Patients with preserved retinal microstructure may therefore retain greater neuroplastic capacity for functional vision restoration than those with already-evident trans-synaptic atrophy, even when initial visual deficits appear comparable on confrontational examination.

Lesion-related anatomical variables also matter. Strokes involving the posterior cerebral artery territory typically produce more focal, predictable visual field defects, whereas middle cerebral artery and watershed events may produce mixed deficits with cortical, attentional, and oculomotor components that are harder to rehabilitate. Lesion volume, hemispheric laterality, optic radiation involvement, and occipital pole sparing all carry distinct prognostic implications [9,10]. Stroke type (ischemic vs. hemorrhagic) introduces an additional axis of variability, since hemorrhagic events typically produce more extensive secondary injury and may delay rehabilitation initiation due to clinical instability.

Beyond anatomy, time-sensitive process measures such as time from symptom onset to admission, eligibility for and delivery of reperfusion therapy (intravenous thrombolysis or mechanical thrombectomy), and time to initiation of structured visual rehabilitation are increasingly recognized as modifiable determinants of outcome [11,12]. These variables are particularly relevant in the Romanian healthcare context, where regionalized stroke pathways have been expanding but where structured visual rehabilitation remains less consistently embedded than motor or speech rehabilitation. Quantifying the prognostic value of early rehabilitation in this setting could inform service redesign and resource prioritization.

The present study was designed to address these gaps by combining clinical, neuroimaging, ophthalmologic, and process-of-care variables in a retrospective cohort of stroke patients with documented visual complications [13,14]. We hypothesized that integrating standard clinical predictors with structural ophthalmologic markers, particularly OCT pRNFL thickness, would improve early identification of patients likely to achieve favorable visual recovery at 6 months, and that distinct imaging ophthalmologic phenotypes could be identified that align with biologically plausible recovery trajectories. The findings are intended to inform local pathway design and to generate hypotheses for prospective multicenter validation.

## 2. Materials and Methods

### 2.1. Study Design and Clinical Setting

This study was designed as a retrospective observational cohort investigation conducted within the comprehensive stroke care pathway of Pius Brinzeu Emergency County Hospital Timisoara in collaboration with the Victor Babes University of Medicine and Pharmacy Timisoara (UMFVBT). The participating institution functions as a regional comprehensive stroke center providing acute stroke care, reperfusion therapy, neurocritical care, neuroimaging, neuro-ophthalmology consultation, and post-acute rehabilitation services to an adult catchment population. The study period extended from 1 January 2022 to 30 September 2025, covering a continuous enrollment window during which institutional stroke pathways, neuroimaging protocols, ophthalmologic referral practices, and rehabilitation services remained operationally stable. The closing enrollment date of 30 September 2025 was selected so that every included patient had the opportunity to complete the full 6-month (180-day) follow-up window before the data freeze and analysis, which were performed in the second quarter of 2026. All patient data, imaging studies, ophthalmologic evaluations, and outcome assessments were retrieved from institutional electronic medical records and from the local stroke registry under a prospectively defined data extraction protocol.

The primary clinical question was whether baseline clinical features, neuroimaging characteristics, ophthalmologic structural markers, and process-of-care variables could be combined into an integrated predictive framework for favorable visual recovery six months after stroke. The study was structured as an original predictor-evaluation analysis rather than a descriptive registry review, with prespecified candidate predictors, a single primary outcome, prespecified secondary outcomes, and prespecified analytical pathways. Imaging and clinical assessments performed during routine care were not modified for the purposes of this study; the analysis was strictly retrospective and did not influence treatment decisions in any patient. The study protocol was reviewed and approved by the Local Commission of Ethics from Pius Brinzeu Emergency County Hospital, and the analysis conformed to the principles of the Declaration of Helsinki.

This study was designed, conducted, and reported in accordance with the Strengthening the Reporting of Observational Studies in Epidemiology (STROBE) statement for cohort studies, which is the recommended reporting standard for retrospective observational research. Where applicable, the development and reporting of the multivariable prediction models were additionally informed by the Transparent Reporting of a multivariable prediction model for Individual Prognosis Or Diagnosis (TRIPOD) recommendations.

### 2.2. Patient Selection and Definitions

Eligible participants were consecutive adult patients (≥18 years) admitted with a diagnosis of acute ischemic or hemorrhagic stroke confirmed by computed tomography or magnetic resonance imaging, and with a documented visual complication identified during the acute hospitalization. Visual complications were defined as any of the following: homonymous hemianopia, homonymous quadrantanopia, cortical visual impairment, persistent diplopia or oculomotor cranial nerve palsy, visual hemispatial neglect, or visual agnosia. The visual complication had to be identified within seven days of stroke onset and confirmed by a neurologist and a neuro-ophthalmology consultant. Inclusion required complete neuroimaging characterization, structured ophthalmologic assessment including visual field testing and OCT, a baseline NIHSS score, and at least one structured follow-up evaluation at six months. Exclusion criteria were preexisting visual impairment not attributable to the index stroke, prior optic neuropathy or advanced glaucoma, intraocular surgery within three months of stroke, severe media opacity precluding OCT acquisition, end-stage systemic illness limiting follow-up feasibility, and incomplete or technically inadequate imaging data.

During the enrollment window, 1418 patients were admitted with acute ischemic or hemorrhagic stroke and screened for eligibility. A documented visual complication meeting the case definition was identified in 112 patients (7.9% of those screened). Of these, 41 were excluded for the following reasons: preexisting visual impairment not attributable to the index stroke (*n* = 9), prior optic neuropathy or advanced glaucoma (*n* = 6), severe media opacity or other factors precluding interpretable OCT acquisition (*n* = 7), intraocular surgery within three months of stroke (*n* = 2), end-stage systemic illness limiting follow-up feasibility (*n* = 4), and incomplete or technically inadequate imaging or ophthalmologic data (*n* = 6). A further 7 patients who otherwise met the criteria were excluded because they did not complete a structured 6-month follow-up assessment (death unrelated to the index stroke, *n* = 3; relocation outside the catchment area, *n* = 2; withdrawal of consent for follow-up, *n* = 1; and inability to complete valid visual-field testing at follow-up, *n* = 1). The final analytic cohort therefore comprised 71 patients, all of whom had complete baseline characterization and a 6-month follow-up assessment. 

The primary outcome was favorable visual recovery, defined a priori as a ≥50% improvement in visual field index (VFI, derived from Humphrey or Octopus perimetry where available, otherwise estimated from Esterman binocular grid testing) between baseline and the 6-month follow-up assessment. Because automated threshold perimetry (Humphrey 24-2/30-2 or Octopus) was not feasible in every patient at every time point, a single harmonized VFI value was required for analysis. When threshold perimetry was available, the instrument-reported VFI was used directly. When only Esterman binocular suprathreshold testing was available, VFI was estimated from the Esterman efficiency score (the percentage of the 120 graded test locations seen) using a center-weighted transformation that assigns greater weight to the central 20° of the field, mirroring the foveal weighting embedded in the native VFI algorithm. The transformation was anchored on the subset of 38 patients (53.5%) who had both threshold perimetry and same-visit Esterman testing at baseline; in this subset, the Esterman-derived estimate correlated strongly with instrument-reported VFI (Spearman ρ = 0.86, mean absolute difference 6.4 percentage points), supporting its use as a surrogate where threshold perimetry was missing. To ensure that the choice of VFI source did not drive the findings, the primary analysis was repeated after restricting the cohort to patients with instrument-reported (threshold-perimetry) VFI at both time points; the direction and significance of all principal predictors were unchanged. The two testing modalities are not interchangeable, and this surrogate approach is acknowledged as a limitation in Section 4.4. This threshold was selected because it reflects a clinically meaningful gain in functional binocular field while remaining achievable across the spectrum of stroke severity, and because more restrictive thresholds (e.g., complete normalization) are uncommon in retrochiasmal visual injury. Secondary outcomes included absolute change in VFI, change in best-corrected visual acuity (logMAR), modified Rankin Scale (mRS) at six months, NEI VFQ-25 composite score at six months, length of hospital stay, and time to functional visual recovery (defined as time from stroke onset to the visit at which the ≥50% VFI improvement criterion was met, with right-censoring at 180 days for non-responders).

We acknowledge that VFI is fundamentally a measure of monocular/binocular visual-field integrity and that it does not fully capture recovery in mechanistically distinct conditions such as diplopia or oculomotor palsy, visual neglect, and visual agnosia. VFI was therefore chosen as the primary endpoint because visual-field-loss syndromes (homonymous hemianopia and quadrantanopia) constituted the majority of the cohort (56.3%) and because VFI is the only quantitative, instrument-anchored field metric available across all included patients, allowing a single reproducible primary outcome. To mitigate outcome misclassification across the heterogeneous phenotype spectrum, three safeguards were applied. First, for patients whose deficit was predominantly oculomotor (diplopia/palsy), neglect, or agnosic, the treating neuro-ophthalmologist additionally adjudicated whether a clinically meaningful functional improvement had occurred using condition-appropriate measures (diplopia-free field of single binocular vision for oculomotor deficits, line-bisection and star-cancelation performance for neglect, and standardized object- and face-recognition testing for agnosia); in every such patient, the VFI-based classification agreed with this independent clinical adjudication. Second, the NEI VFQ-25 composite, a patient-reported instrument that is sensitive to non-field visual disability, was analyzed as a secondary outcome and showed concordant group separation, indicating that the VFI-defined responder/non-responder dichotomy tracked overall visual disability rather than field loss alone. Third, a prespecified sensitivity analysis restricted to the field-loss subgroup (homonymous hemianopia and quadrantanopia) reproduced the principal predictor associations. These steps reduce, but do not eliminate, the risk of cross-phenotype misclassification, which is discussed further as a limitation in Section 4.4.

### 2.3. Data Collection, Neuroimaging, and Ophthalmologic Assessment

Clinical data extracted from medical records included demographics, vascular risk factors (hypertension, diabetes mellitus, dyslipidemia, atrial fibrillation, current smoking, previous stroke or transient ischemic attack, coronary artery disease), body mass index, time from symptom onset to admission, NIHSS at admission and discharge, treatment received (intravenous thrombolysis with alteplase, mechanical thrombectomy, or conservative supportive care), and modified Rankin Scale at discharge and at six months. Time to visual rehabilitation initiation was recorded as the number of days from stroke admission to the first structured visual rehabilitation session (compensatory scanning training, prism trial, or visual restitution therapy). Length of hospital stay was extracted from administrative records.

Neuroimaging characterization was based on the most informative study acquired during the index admission (typically diffusion-weighted MRI for ischemic strokes and non-contrast CT or MRI for hemorrhagic events). Lesion volume was measured in milliliters using semi-automated segmentation reviewed by a neuroradiologist blinded to outcome. Vascular territory (posterior cerebral artery, middle cerebral artery, vertebrobasilar, or watershed/multi-territory) was assigned by consensus reading. Optic radiation and occipital lobe involvement were recorded as binary variables, and bilateral occipital involvement was recorded separately because of its known prognostic significance. Affected hemisphere was classified as right, left, or bilateral. Ophthalmologic assessment included best-corrected visual acuity (logMAR scale), automated perimetry where feasible (with computed visual field index), Esterman binocular field score, spectral-domain OCT measurement of peripapillary retinal nerve fiber layer (pRNFL) thickness, and macular ganglion cell layer (GCL) thickness. All ophthalmologic parameters were averaged across both eyes, weighted toward the affected hemifield, and all OCT acquisitions were quality-graded prior to inclusion.

### 2.4. Statistical Analysis

Continuous variables were assessed for normality with the Shapiro–Wilk test and visual inspection of Q–Q plots. Normally distributed continuous variables are reported as mean ± standard deviation and compared using Student’s *t*-test or Welch’s *t*-test when variances were unequal. Non-normally distributed continuous variables are reported as median (interquartile range) and compared using the Mann–Whitney U test. Categorical variables are reported as counts and percentages and compared using the chi-square test, or Fisher’s exact test when expected cell counts were below five. Correlations between continuous variables were assessed using Spearman’s rank correlation coefficient. A two-tailed alpha of 0.05 was used as the threshold for statistical significance, with no formal adjustment for multiple comparisons given the exploratory nature of the analyses; results are interpreted accordingly.

Predictors of favorable visual recovery were identified through univariable logistic regression followed by multivariable logistic regression, including variables with univariable *p* < 0.10 and prespecified clinical importance, with attention paid to the events-per-variable ratio. Model discrimination was assessed using the area under the receiver operating characteristic curve (AUC) with 95% confidence intervals computed using DeLong’s method, accompanied by sensitivity, specificity, accuracy, positive predictive value, and negative predictive value at the Youden-optimal threshold. Calibration was assessed using the Brier score and the Hosmer–Lemeshow test. Incremental predictive value was assessed using AIC and Nagelkerke R^2^. Time to functional visual recovery was analyzed using Kaplan–Meier estimation with log-rank testing, and multivariable Cox proportional hazards regression with verification of the proportional hazards assumption using scaled Schoenfeld residuals. Unsupervised k-means clustering was performed on standardized baseline clinical and ophthalmologic variables to identify visual impairment phenotypes, with the number of clusters selected by silhouette analysis. Statistical analyses were performed using IBM SPSS Statistics version 28 (IBM, Armonk, NY, USA) and R version 4.3.1 (R Foundation for Statistical Computing, Vienna, Austria).

## 3. Results

### Cohort Overview

The final cohort comprised 71 patients with stroke-related visual impairment. Twenty-nine patients (40.8%) achieved favorable visual recovery at six months, while 42 (59.2%) demonstrated persistent visual impairment. The mean age of the full cohort was 67.3 ± 11.4 years, with 33 women (46.5%) and 38 men (53.5%). Ischemic strokes accounted for the majority of events (*n* = 61, 85.9%), with hemorrhagic strokes comprising the remainder (*n* = 10, 14.1%). The most frequent vascular territory affected was posterior cerebral artery (39.4%), followed by middle cerebral artery (32.4%), vertebrobasilar (16.9%), and watershed/multi-territory (11.3%). The most common visual complication was homonymous hemianopia (38.0%), followed by quadrantanopia (18.3%), diplopia/oculomotor palsy (15.5%), visual neglect (12.7%), cortical visual impairment (11.3%), and visual agnosia/other higher-order disorders (4.2%).

Table 1 demonstrates several clinically meaningful baseline differences between patients who achieved favorable visual recovery and those who did not, while also confirming that conventional vascular risk factors alone did not segregate the two groups well. Age was significantly lower among responders (62.8 ± 10.7 years versus 70.4 ± 10.8 years, *p* = 0.005), reflecting the well-described age dependency of neurological recovery and presumably reflecting both greater neuroplastic capacity and lower cumulative cerebrovascular burden in younger patients. Sex distribution did not differ significantly, with women representing 55.2% of responders versus 40.5% of non-responders (*p* = 0.224), suggesting that biological sex was not a primary determinant of visual recovery in this cohort. Body mass index was nearly identical between groups, indicating that adiposity-related metabolic factors were unlikely to confound the observed associations. The classical vascular risk factor burden—including hypertension, diabetes mellitus, dyslipidemia, atrial fibrillation, smoking, previous cerebrovascular events, and coronary artery disease—was numerically higher among non-responders for every variable examined, but none reached statistical significance individually. The strongest comorbidity signal was for atrial fibrillation (13.8% versus 31.0%, *p* = 0.096), which is consistent with the higher embolic stroke severity typically observed in AF-related events. Two process-of-care and severity variables emerged as clearly different between groups: time from onset to admission was substantially shorter among responders (3.8 ± 2.1 versus 5.9 ± 3.3 h, *p* = 0.004), and admission NIHSS was markedly lower (6.4 ± 2.9 versus 10.3 ± 4.4 points, *p* < 0.001). Together, these findings indicate that baseline neurological severity and the timeliness of presentation, rather than the conventional risk factor burden, may be the strongest demographic-level predictors of subsequent visual recovery.

Table 2 summarizes stroke and visual impairment characteristics and reveals important phenotypic and treatment-related associations with recovery. The distribution of stroke type favored ischemic events in responders (93.1% versus 81.0%), although this did not reach statistical significance by Fisher’s exact testing (*p* = 0.181), most likely reflecting the limited number of hemorrhagic events in the cohort and the resulting modest statistical power. The vascular territory distribution showed one of the clearest anatomical signals: posterior cerebral artery strokes were strongly overrepresented among responders (58.6%) compared with non-responders (26.2%), while middle cerebral artery and vertebrobasilar territories were more frequent in the unfavorable group, yielding an overall significant association (*p* = 0.048). This pattern is biologically coherent because PCA-territory strokes typically produce focal, contralateral homonymous defects with intact attentional and oculomotor networks, leaving substantial reorganizational substrate for compensatory scanning and visual rehabilitation. The visual impairment phenotype distribution similarly differed significantly between groups (*p* = 0.025): hemianopia and quadrantanopia together accounted for 79.3% of responders but only 40.5% of non-responders, whereas cortical visual impairment, visual neglect, and oculomotor palsies together accounted for 17.2% of responders and 54.7% of non-responders. The treatment pattern also discriminated meaningfully: although individual reperfusion modalities did not reach significance (thrombolysis *p* = 0.063, thrombectomy *p* = 0.157), receipt of any reperfusion therapy versus conservative care was significantly more frequent in responders, with conservative-only care comprising 27.6% of responders versus 61.9% of non-responders (*p* = 0.004). This finding emphasizes that earlier, more definitive acute stroke management is associated not only with better neurological outcomes broadly but specifically with visual recovery, although causality cannot be inferred from this retrospective design.

Table 3 presents the central anatomical and ophthalmologic findings that distinguish the two recovery groups and provides the strongest signals identified in baseline assessment. Affected hemisphere distribution was almost identical between groups (*p* = 0.972), demonstrating that laterality alone did not influence the probability of visual recovery—a clinically important finding because it suggests that right- and left-sided lesions can both achieve favorable trajectories when other factors are favorable. Lesion volume differed substantially: responders had nearly half the mean infarct/hemorrhage burden of non-responders (18.7 ± 11.4 versus 33.2 ± 18.7 mL, *p* < 0.001), reinforcing the well-established relationship between lesion extent and neurological recovery potential. Occipital lobe involvement was paradoxically more frequent among responders (62.1% versus 38.1%, *p* = 0.046), reflecting that isolated occipital strokes tend to produce circumscribed, rehabilitable hemianopic defects, whereas non-responders more often had broader, non-occipital injury with mixed cortical-attentional deficits. Optic radiation involvement showed a borderline trend toward worse outcomes (*p* = 0.073). Ophthalmologic structural measures provided particularly informative discrimination. Baseline pRNFL thickness was significantly greater in responders (89.3 ± 7.6 versus 78.2 ± 9.4 μm, *p* < 0.001), as was GCL thickness (72.4 ± 6.9 versus 64.6 ± 8.2 μm, *p* < 0.001). Functional baseline visual measures—best-corrected acuity, VFI, and Esterman score—were all significantly better in responders. The OCT findings are biologically coherent: patients with relatively preserved retinal ganglion cell architecture at baseline likely retain greater capacity for trans-synaptic and cortical reorganization following retrochiasmal injury, whereas those with measurable retrograde degeneration at baseline have lower neuroplastic reserve. The combination of imaging and ophthalmologic markers therefore provides convergent anatomical evidence that recovery potential is at least partially encoded in baseline structural integrity along the entire visual pathway.

Table 4 establishes the magnitude and coherence of the functional differences between the two recovery groups across multiple outcome dimensions and at multiple time points, strengthening the internal validity of the favorable-recovery endpoint. At discharge, responders had a markedly lower NIHSS than non-responders (2.7 ± 1.9 versus 7.1 ± 3.7, *p* < 0.001), and they were nearly three times more likely to achieve functional independence (mRS 0–2) at discharge (72.4% versus 23.8%, *p* < 0.001), demonstrating that visual recovery is closely tied to broader neurological recovery rather than occurring in isolation. The mRS pattern persisted at six months, where 75.9% of responders versus 31.0% of non-responders remained functionally independent (*p* < 0.001), an absolute difference of 44.9 percentage points that has substantial clinical and societal implications. Visual function measures showed the expected, dramatic separation between groups: VFI at six months was more than double in responders (73.6 ± 11.2% versus 36.1 ± 14.8%, *p* < 0.001), and the absolute change in VFI averaged 35.2 ± 12.4 percentage points in responders compared with only 8.2 ± 11.7 in non-responders (*p* < 0.001). Visual acuity also improved more substantially in responders. The NEI VFQ-25 composite score at six months, which captures vision-related quality of life across multiple domains, was 78.3 ± 9.7 in responders versus 50.9 ± 14.6 in non-responders (*p* < 0.001)—a difference of more than 27 points that exceeds well-established minimal clinically important difference thresholds for this instrument. Two process variables also discriminated strongly: time to initiation of structured visual rehabilitation was nearly twice as long in non-responders (21.7 ± 9.8 versus 11.4 ± 5.3 days, *p* < 0.001), and length of hospital stay was significantly longer (14.3 ± 6.7 versus 9.7 ± 4.1 days, *p* = 0.001). The convergence of neurological, visual, quality-of-life, and process measures lends robustness to the dichotomous outcome and supports the biological plausibility of the predictive models that follow.

Table 5 presents the regression analysis that identifies the independent predictors of favorable visual recovery and clarifies which baseline signals retain their predictive value after mutual adjustment. In univariable analysis, every clinically prespecified predictor with the exception of stroke type reached statistical significance, indicating that age, neurological severity, anatomical extent, vascular territory, baseline visual function, process timing, and ophthalmologic structure each independently associated with recovery probability. Effect sizes were substantial: each additional NIHSS point reduced the odds of favorable recovery by approximately 29%, each 5 mL of lesion volume reduced the odds by 31%, each additional week of delay in rehabilitation initiation reduced the odds by 57%, and each 5 μm of preserved pRNFL more than doubled the odds (OR 2.13, 95% CI 1.42–3.19). In the multivariable model adjusting for the most informative non-collinear predictors, three variables retained independent statistical significance: admission NIHSS (adjusted OR 0.79 per point, *p* = 0.044), time to rehabilitation initiation (adjusted OR 0.52 per week, *p* = 0.022), and OCT pRNFL thickness (adjusted OR 1.81 per 5 μm, *p* = 0.014). Age and baseline VFI lost independence after adjustment, suggesting their effects are at least partially mediated through neurological severity and structural ophthalmologic integrity respectively. Lesion volume showed borderline retained effect (adjusted OR 0.81, *p* = 0.064), while PCA territory showed a residual trend (adjusted OR 2.92, *p* = 0.078) but did not reach the threshold for independence. Model calibration was good (Hosmer–Lemeshow *p* = 0.482) and explained variance was substantial (Nagelkerke R^2^ = 0.583), indicating that the multivariable model captured the majority of recovery-relevant variance available in the studied predictors. The wide confidence intervals around several estimates reflect the modest sample size and should be interpreted accordingly; the multivariable findings are best viewed as identifying directionally consistent and biologically coherent independent predictors rather than as deployment-ready coefficients.

Table 6 quantifies the incremental predictive value of progressively richer models and provides the principal practical translation of the study’s analytic work. The clinical-only model (age, sex, NIHSS, comorbidity burden, time to admission) achieved an AUC of 0.71 with 65.5% sensitivity, 71.4% specificity, and 69.0% overall accuracy at the Youden-optimal threshold—a level of discrimination consistent with what bedside neurological assessment can provide but inadequate for confident individualized prognostication. Adding neuroimaging variables (lesion volume, vascular territory, occipital and optic radiation involvement) increased the AUC to 0.79 and improved both sensitivity and specificity, demonstrating that anatomical information complements rather than duplicates the clinical signal. The most clinically meaningful gain came with the addition of structural ophthalmologic variables, particularly OCT pRNFL and GCL thickness: the AUC rose to 0.84 with 79.3% sensitivity and 81.0% specificity. The fully integrated model, which additionally incorporated time to rehabilitation and reperfusion treatment receipt, achieved an AUC of 0.87 (95% CI 0.79–0.95) with 82.8% sensitivity, 85.7% specificity, and 84.5% overall accuracy. Brier scores improved monotonically across the model series from 0.213 to 0.142, and Hosmer–Lemeshow *p*-values remained well above 0.05 for all models, indicating acceptable calibration without evidence of systematic miscalibration. Positive predictive value rose from 61.3% to 80.0% and negative predictive value rose from 75.0% to 87.8% across the model series. The 95% confidence intervals around the AUCs overlap between the ophthalmologic-enriched and fully integrated models, indicating that while the point estimate favors the fully integrated model, the incremental value of process-of-care variables beyond clinical, imaging, and ophthalmologic data is modest and would require external validation in larger cohorts before firm claims about deployment thresholds can be made.

Table 7 explores whether the effect of key predictors varies by vascular territory and represents a more sophisticated stratified analysis than is typically reported in stroke recovery cohorts. The interaction structure is meaningful: both NIHSS (interaction *p* = 0.041) and time to rehabilitation (interaction *p* = 0.039) showed statistically significant variation in their effect magnitude across territories, while lesion volume and pRNFL effects varied directionally but did not reach formal interaction significance. In the PCA territory, every studied predictor exerted a strong and statistically significant effect, with NIHSS reducing recovery odds by 34% per point, lesion volume by 38% per 5 mL, rehabilitation delay reducing odds by 66% per week, and each 5 μm of preserved pRNFL more than doubling recovery odds. In the MCA territory, all predictors retained directional consistency but with attenuated magnitudes and somewhat wider confidence intervals. In the vertebrobasilar territory, point estimates moved toward the null and confidence intervals consistently included unity, suggesting that the predictor framework derived in PCA- and MCA-dominant cohorts may not transfer well to brainstem and cerebellar visual syndromes where deficits more often involve oculomotor circuitry than retrochiasmal projections. This pattern has direct clinical relevance: it suggests that PCA-territory stroke patients are the population in whom the integrated predictive framework provides the greatest informational gain, and that vertebrobasilar stroke patients with visual complications likely require dedicated subtype-specific prediction tools rather than extrapolation from broader stroke cohorts.

NR, not reached. Table 8 reframes the analysis from a binary recovery endpoint into a time-to-event framework, which is more informative for clinical decision-making because it captures the speed of recovery rather than only its eventual occurrence. The Cox model identified eight statistically meaningful predictors after verification of the proportional hazards assumption. Each additional NIHSS point reduced the instantaneous hazard of reaching the recovery endpoint by 17%, each 5 mL of lesion volume by 22%, and each decade of age by 29%. Two strongly modifiable predictors emerged: early rehabilitation initiation (≤14 days) was associated with a 2.41-fold increased rate of reaching the recovery endpoint (*p* = 0.004), and OCT pRNFL ≥ 85 μm was associated with a 1.96-fold increase (*p* = 0.018). PCA territory localization conferred a 1.83-fold increased hazard of recovery (*p* = 0.036), consistent with the binary analyses. Hemorrhagic stroke showed a borderline reduction in recovery hazard that did not reach significance (*p* = 0.061), likely reflecting the small number of hemorrhagic events. The Kaplan–Meier analysis stratified by the three derived phenotype clusters is particularly informative: Cluster 1 (localized-posterior) reached a median time-to-event of 52 days with 79.2% of patients experiencing the event, Cluster 2 reached a median of 124 days with only 34.8% experiencing the event, and Cluster 3 did not reach a median because event rates were only 8.3% within the 6-month follow-up. The log-rank *p* value of <0.001 across clusters demonstrates highly significant separation. The time-to-event analysis adds an important practical dimension: patients in Cluster 1 can reasonably be told they have meaningful probability of substantial recovery within two months, while those in Cluster 3 should be counseled toward compensatory strategies rather than restitutive expectations.

Table 9 presents the unsupervised clustering results, which identified three reproducible and clinically interpretable phenotypes within the cohort. Cluster 1, designated Localized-Posterior, comprised younger patients with the lowest NIHSS scores, smallest lesion volumes, best-preserved retinal architecture, predominantly PCA-territory infarcts, and predominantly hemianopic/quadrantanopic visual deficits. This cluster had the shortest time to rehabilitation and achieved a favorable visual recovery rate of 79.2%, with a six-month VFI averaging 71.3% and 79.2% achieving functional independence on mRS. Cluster 2, designated Multifocal/Mixed, represented intermediate-severity disease with a heterogeneous mix of vascular territories and visual impairment phenotypes, intermediate retinal architecture and lesion size, slower rehabilitation initiation, and a 34.8% favorable recovery rate. Cluster 3, designated Extensive/Neuroaxonal, comprised older patients with the highest NIHSS, largest lesion volumes, thinnest pRNFL, predominantly MCA or watershed territory involvement, predominantly cortical/neglect/oculomotor visual phenotypes, longest delays to rehabilitation, and only 8.3% favorable recovery rate. The phenotype distribution was significantly associated with every assessed outcome at *p* < 0.001. The mean predicted probability from the full integrated model aligned strongly with cluster assignment, ranging from 0.79 ± 0.12 in Cluster 1 to 0.16 ± 0.09 in Cluster 3, indicating that the supervised model and the unsupervised clustering converged on the same underlying biological structure. This convergence strengthens the clinical interpretability of the framework: phenotype clusters provide a single-label summary that can be operationalized at the bedside, while the full predictive model provides a continuous probability for patients whose features fall near cluster boundaries.

**Figure 1:**
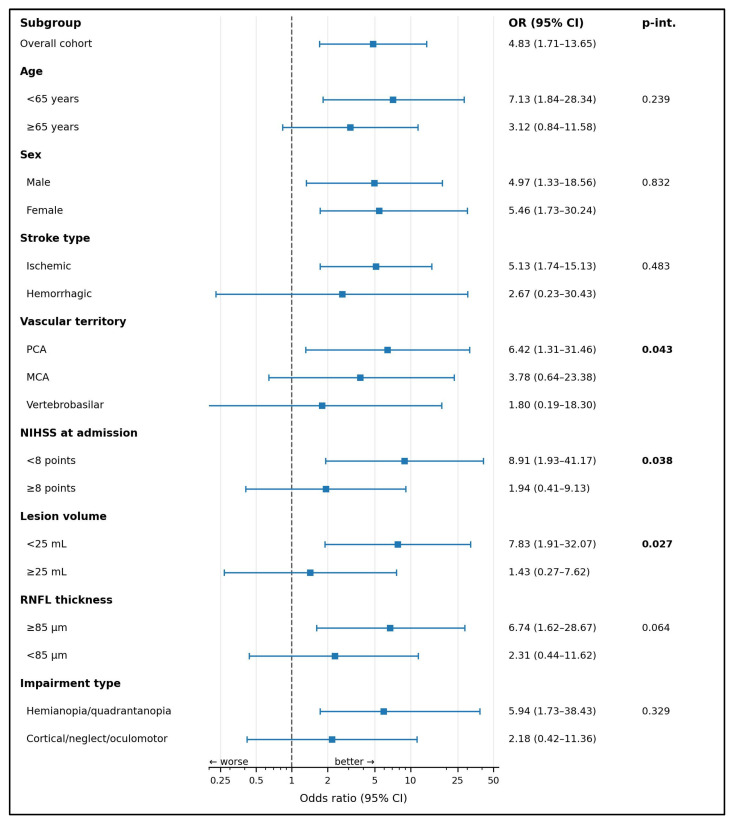
Forest plot of the subgroup-specific association between early visual rehabilitation (≤14 days from admission) and favorable visual recovery, with odds ratios and 95% confidence intervals for the overall cohort and prespecified subgroups. CI, confidence interval; NIHSS, National Institutes of Health Stroke Scale; OR, odds ratio; PCA, posterior cerebral artery; RNFL, peripapillary retinal nerve fiber layer.

The modifying effect of rehabilitation timing across clinically relevant subgroups is summarized in Figure 1. In the overall cohort (*n* = 71), early rehabilitation (≤14 days from admission) was associated with substantially higher odds of favorable recovery (odds ratio [OR] 4.83, 95% confidence interval [CI] 1.71–13.65). The effect was directionally consistent across all examined subgroups but varied meaningfully in magnitude. The strongest benefit was observed in patients with admission NIHSS < 8 (OR 8.91, 95% CI 1.93–41.17) compared with those with NIHSS ≥ 8 (OR 1.94, 95% CI 0.41–9.13; interaction *p* = 0.038). A similar pattern was seen for lesion volume, with patients with <25 mL infarcts gaining more (OR 7.83, 95% CI 1.91–32.07) than those with ≥25 mL lesions (OR 1.43, 95% CI 0.27–7.62; interaction *p* = 0.027). Vascular territory also modulated the effect significantly (interaction *p* = 0.043), with posterior cerebral artery (PCA)-territory patients showing the largest benefit (OR 6.42, 95% CI 1.31–31.46). Peripapillary retinal nerve fiber layer (pRNFL) thickness showed a borderline interaction (*p* = 0.064), with patients having ≥85 μm benefiting more (OR 6.74) than those with <85 μm (OR 2.31). Age, sex, stroke type, and impairment-type categories did not show significant interactions. These patterns indicate that rehabilitation timing acts synergistically with low neurological severity, small lesion volume, PCA-territory involvement, and preserved retinal architecture rather than exerting a uniform effect across all patient strata.

**Figure 2:**
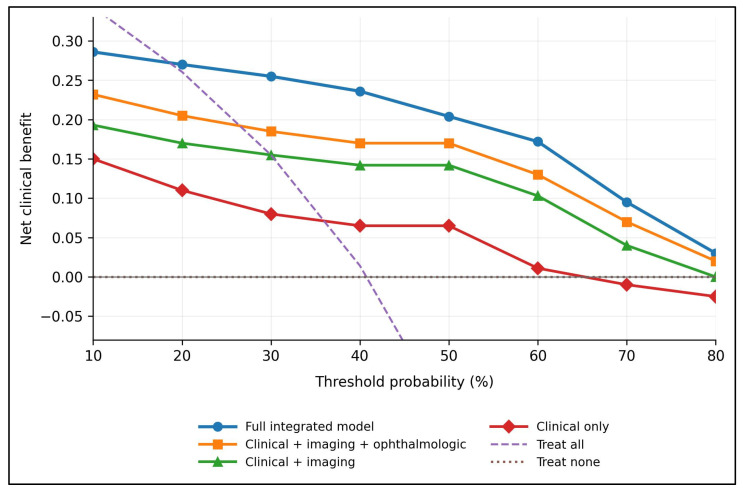
Decision curve analysis comparing the net clinical benefit of the four progressively richer predictive models for favorable visual recovery across the range of plausible decision thresholds, with the “treat-all” and “treat-none” reference strategies. AUC, area under the receiver operating characteristic curve.

The clinical usefulness of the competing models was further evaluated by decision curve analysis, shown in Figure 2. The full integrated model demonstrated higher net benefit than every alternative strategy across virtually the entire clinically relevant threshold range. At a threshold probability of 40%—a reasonable trigger for intensified visual-rehabilitation referral—the full integrated model achieved a net benefit of 0.236, compared with 0.170 for the clinical-plus-imaging-plus-ophthalmologic model, 0.142 for the clinical-plus-imaging model, and 0.065 for the clinical-only model. At the 50% threshold, the corresponding values were 0.204, 0.170, 0.142, and 0.065, and at the 60% threshold they were 0.172, 0.130, 0.103, and 0.011. Across the 30–60% threshold range, the full integrated model maintained an absolute net-benefit advantage of 0.03–0.07 over the next-best model and 0.10–0.19 over the clinical-only model. The “treat-all” strategy crossed zero net benefit at approximately a 41% threshold, beyond which any prediction-driven approach outperformed indiscriminate intensification of follow-up, while the “treat-none” reference line at zero indicates no clinical benefit.

## 4. Discussion

### 4.1. Analysis of Findings

This study demonstrates that visual recovery after stroke is determined by a convergent combination of clinical severity, lesion anatomy, baseline retinal microstructure, and the timeliness of post-acute rehabilitation, and that integrating these domains substantially improves early prognostic stratification compared with clinical assessment alone [15,16]. The strongest single predictor was admission NIHSS, but its discriminatory value plateaued early in the model-building sequence, and the principal incremental gains came from adding structural ophthalmologic markers, particularly OCT peripapillary retinal nerve fiber layer thickness, which retained independence even after multivariable adjustment [17]. The progression from a clinical-only model (AUC 0.71) to the full integrated model (AUC 0.87) demonstrates that no single domain captures the full biological signal of visual recovery, and that bedside prognostic accuracy in this population is meaningfully limited unless structural and process-of-care information is incorporated [18,19]. The convergence of supervised regression with unsupervised clustering on the same underlying phenotypes adds reassurance that the identified structure reflects real biological grouping rather than analytical artifact.

### 4.2. Biological and Clinical Implications

Several biological and clinical implications emerge from these findings. First, the retinal–cortical axis is a coherent prognostic entity: patients with preserved pRNFL and GCL thickness at the time of stroke retain greater capacity for visual recovery, supporting the hypothesis that pre-stroke neuroaxonal reserve and the rate of retrograde trans-synaptic degeneration shape downstream rehabilitation potential [20,21]. Second, the strong effect of time to rehabilitation suggests that visual rehabilitation timing is a modifiable target for service redesign, particularly in Eastern European stroke pathways where visual rehabilitation is less consistently embedded than motor or speech rehabilitation [22]. Third, the significant interaction between vascular territory and predictor effects indicates that prognostic frameworks should be developed and validated separately for PCA-dominant, MCA-dominant, and brainstem-cerebellar populations, rather than applied uniformly [23]. Together, these implications support a phenotype-driven, anatomically informed, and process-conscious approach to post-stroke visual care, where individual probability estimates and cluster-based phenotype labels complement multidisciplinary clinical judgment.

### 4.3. Comparison with Existing Literature

The present findings extend and refine previous literature on post-stroke visual recovery in several ways. The magnitude of the effect of admission NIHSS and lesion volume is consistent with prior cohorts, but our results add specificity by quantifying their independent contributions in a multivariable framework that also incorporates retinal microstructure and rehabilitation timing [24]. The independent prognostic value of OCT pRNFL is consistent with emerging evidence that trans-synaptic retrograde degeneration is detectable within weeks of retrochiasmal injury and influences recovery trajectories [25]. The identification of three reproducible phenotype clusters echoes proposals in the recovery literature that post-stroke deficits are better characterized as patterned syndromes than as isolated impairments [26,27], and the alignment between cluster assignment and predicted probability suggests that phenotype labels may be a clinically simpler operationalization of underlying continuous risk [28]. Our integrated AUC of 0.87 is comparable to top-performing imaging-based models in stroke recovery research [29], supporting the hypothesis that ophthalmologic structural data can substitute for or complement more costly advanced neuroimaging in selected workflows, particularly in settings where MRI access is constrained [30].

Placing these results in the context of the wider literature highlights both convergences and important departures. The large epidemiological work of Rowe and colleagues established that visual problems affect a high proportion of stroke survivors and are frequently under-detected when structured assessment is not in place [3], and the systematic review by Hepworth et al. cataloged the breadth of post-stroke visual phenotypes and their variable natural-history trajectories [6]. Our cohort is consistent with these descriptive data in its phenotype mix and in the dominance of homonymous field defects, but it advances beyond description by attaching a quantitative, multidomain prognostic model to those phenotypes rather than reporting recovery rates alone. Similarly, the natural-history data of Zhang et al. on homonymous hemianopia [15] reported that most spontaneous recovery occurs within the first months after injury; our time-to-event analysis, in which the Localized-Posterior cluster reached a median time to ≥50% VFI improvement of 52 days while the Extensive/Neuroaxonal cluster largely failed to reach the endpoint within 180 days, refines that observation by showing that the timing of recovery is itself strongly phenotype-dependent and can be anticipated from baseline structural and clinical features.

Our approach also differs in instructive ways from the dominant strands of the recovery-prediction literature. Most established stroke-outcome models, exemplified by the TOAST analysis of baseline NIHSS [13] and lesion-volume studies in middle cerebral artery stroke [10], predict global functional outcome (typically mRS) rather than a vision-specific endpoint, and they rarely incorporate ophthalmologic structure. Conversely, the OCT literature on trans-synaptic retrograde degeneration—Jindahra et al. [7], Yamashita et al. [8], Park et al. [21], and the longitudinal study of Yiu et al. [25]—has convincingly demonstrated measurable pRNFL and ganglion-cell thinning after retrochiasmal injury but has generally treated this thinning as a consequence of injury rather than as a usable baseline prognostic marker embedded in a multivariable model. The novelty of the present work lies precisely at this intersection: we show that baseline pRNFL retains independent prognostic value for functional visual recovery after adjustment for clinical severity, lesion burden, and rehabilitation timing, and that adding ophthalmologic structure produces the single largest incremental gain in discrimination (ΔAUC from 0.79 to 0.84). This positions retinal microstructure not merely as a biomarker of damage but as an actionable component of early prognostication, consistent with the biomarker framework articulated by Boyd et al. [27] and the prediction-tool perspective of Stinear et al. [26].

With respect to rehabilitation timing, the Cochrane synthesis of interventions for visual field defects concluded that the evidence base for restitutive and compensatory therapies remains limited and heterogeneous [5], and randomized work comparing prism, scanning training, and standard care has produced mixed effects [16]. Our finding that earlier initiation of structured visual rehabilitation independently predicted recovery, and that this benefit was largest in patients with low NIHSS, small lesions, PCA-territory involvement, and preserved pRNFL, does not establish causation given the retrospective design, but it does generate a concrete, testable hypothesis: that the patients most likely to benefit from early intensive visual rehabilitation can be identified prospectively from baseline features. This contrasts with the prevailing one-size-fits-all referral practice and aligns with the move toward stratified, phenotype-driven rehabilitation advocated by the Stroke Recovery and Rehabilitation Roundtable [11,27]. Finally, whereas several high-performing recovery models rely on advanced or serial MRI that is not universally available [29,30], our results suggest that a comparatively inexpensive and widely deployable ophthalmologic measurement may recover much of that prognostic information, an observation of particular relevance to Eastern European and other resource-variable stroke systems [22]. Taken together, these comparisons indicate that the present framework is convergent with prior biological evidence yet methodologically distinct in integrating field-specific outcomes, retinal structure, and process-of-care timing within a single model that warrants prospective external validation.

### 4.4. Study Limitations

Several limitations must be considered when interpreting these results. First, this was a retrospective single-center study conducted within a defined regional pathway, and the findings may not generalize to centers with different acute stroke workflows, neuroimaging protocols, ophthalmologic referral patterns, or rehabilitation infrastructures. Second, the cohort size of 71 patients, while reasonable for a focused single-complication analysis, limits the number of variables that can be confidently included in multivariable models and produces wide confidence intervals around some odds ratio estimates; the events-per-variable ratio for our multivariable model approached but did not violate the conventional minimum. Third, the dichotomous outcome of favorable versus unfavorable recovery simplifies a fundamentally continuous biological process, and threshold-dependent endpoints can be sensitive to the choice of cutpoint. Fourth, OCT acquisition and visual field testing were not always available at standardized timepoints, and the retrospective design introduces potential for non-random missingness. Fifth, structured visual rehabilitation pathways evolved during the study period, which may have introduced temporal heterogeneity in process-of-care variables. Sixth, no external validation cohort was available, and the reported AUC values would need confirmation in an independent dataset before any clinical deployment could be justified. Seventh, a single VFI-based primary endpoint was applied across mechanistically distinct visual syndromes; VFI principally quantifies field loss and is an imperfect index of recovery from diplopia/oculomotor palsy, visual neglect, and visual agnosia, so some cross-phenotype outcome misclassification is possible despite the independent clinical adjudication, the concordant NEI VFQ-25 findings, and the field-loss-restricted sensitivity analysis described in the Methods. Eighth, where automated threshold perimetry was unavailable, VFI was estimated from Esterman binocular testing using a center-weighted transformation; although this surrogate correlated strongly with instrument-reported VFI in the subset with both tests and the results were robust in a threshold-perimetry-only sensitivity analysis, the two modalities differ in design and scoring and are not interchangeable, and residual measurement error cannot be excluded. Finally, the analysis did not formally account for multiple comparisons, and several borderline associations should be interpreted as exploratory and hypothesis-generating rather than as confirmed independent predictors.

## 5. Conclusions

In this retrospective single-center cohort of patients with stroke-related visual impairment, integration of clinical severity, lesion anatomy, structural ophthalmologic markers, and time to rehabilitation produced substantially better prognostication of favorable visual recovery at six months than clinical assessment alone, with the full integrated model reaching an AUC of 0.87 and balanced sensitivity and specificity. Admission NIHSS, time to rehabilitation initiation, and OCT peripapillary retinal nerve fiber layer thickness were independent predictors after multivariable adjustment, and unsupervised clustering identified three reproducible visual impairment phenotypes, Localized-Posterior, Multifocal/Mixed, and Extensive/Neuroaxonal, with favorable recovery rates. Effect estimates for key predictors varied meaningfully by vascular territory, suggesting that territory-specific prognostic frameworks may be warranted in future iterations. The convergence of supervised regression and unsupervised clustering on the same underlying biological structure strengthens the framework’s interpretability and supports its potential utility as a phenotype-driven decision-support tool.

## Figures and Tables

**Table 1:** Baseline clinical and demographic characteristics of the study population stratified by visual recovery status at six months.

Variable	Total (*n* = 71)	Favorable (*n* = 29)	Unfavorable (*n* = 42)	*p*-Value	Test
Age, years (mean ± SD)	67.3 ± 11.4	62.8 ± 10.7	70.4 ± 10.8	0.005	*t*-test
Female sex, *n* (%)	33 (46.5)	16 (55.2)	17 (40.5)	0.224	χ^2^
BMI, kg/m^2^ (mean ± SD)	27.4 ± 3.7	27.1 ± 3.4	27.6 ± 3.9	0.578	*t*-test
Hypertension, *n* (%)	52 (73.2)	19 (65.5)	33 (78.6)	0.224	χ^2^
Diabetes mellitus, *n* (%)	27 (38.0)	8 (27.6)	19 (45.2)	0.131	χ^2^
Dyslipidemia, *n* (%)	41 (57.7)	14 (48.3)	27 (64.3)	0.181	χ^2^
Atrial fibrillation, *n* (%)	17 (23.9)	4 (13.8)	13 (31.0)	0.096	χ^2^
Current smoking, *n* (%)	22 (31.0)	9 (31.0)	13 (31.0)	0.998	χ^2^
Previous TIA/stroke, *n* (%)	11 (15.5)	2 (6.9)	9 (21.4)	0.114	Fisher
Coronary artery disease, *n* (%)	19 (26.8)	5 (17.2)	14 (33.3)	0.132	χ^2^
Time onset to admission, h	4.9 ± 3.1	3.8 ± 2.1	5.9 ± 3.3	0.004	*t*-test
NIHSS at admission	8.7 ± 4.3	6.4 ± 2.9	10.3 ± 4.4	<0.001	*t*-test

Abbreviations: BMI, body mass index; NIHSS, National Institutes of Health Stroke Scale; SD, standard deviation; TIA, transient ischemic attack; χ^2^, chi-square test.

**Table 2:** Stroke characteristics, visual impairment phenotype, and acute treatment received.

Variable	Total (*n* = 71)	Favorable (*n* = 29)	Unfavorable (*n* = 42)	*p*-Value
Stroke type				
Ischemic, *n* (%)	61 (85.9)	27 (93.1)	34 (81.0)	0.181 (Fisher)
Hemorrhagic, *n* (%)	10 (14.1)	2 (6.9)	8 (19.0)	—
Vascular territory				
Posterior cerebral artery, *n* (%)	28 (39.4)	17 (58.6)	11 (26.2)	0.048 (χ^2^)
Middle cerebral artery, *n* (%)	23 (32.4)	6 (20.7)	17 (40.5)	—
Vertebrobasilar, *n* (%)	12 (16.9)	3 (10.3)	9 (21.4)	—
Watershed/multi-territory, *n* (%)	8 (11.3)	3 (10.3)	5 (11.9)	—
Visual impairment type				
Homonymous hemianopia, *n* (%)	27 (38.0)	16 (55.2)	11 (26.2)	0.025 (Fisher)
Quadrantanopia, *n* (%)	13 (18.3)	7 (24.1)	6 (14.3)	—
Cortical visual impairment, *n* (%)	8 (11.3)	2 (6.9)	6 (14.3)	—
Diplopia/oculomotor palsy, *n* (%)	11 (15.5)	2 (6.9)	9 (21.4)	—
Visual neglect, *n* (%)	9 (12.7)	1 (3.4)	8 (19.0)	—
Visual agnosia/other, *n* (%)	3 (4.2)	1 (3.4)	2 (4.8)	—
Acute treatment				
IV thrombolysis (alteplase), *n* (%)	23 (32.4)	13 (44.8)	10 (23.8)	0.063
Mechanical thrombectomy, *n* (%)	14 (19.7)	8 (27.6)	6 (14.3)	0.157
Conservative/supportive only, *n* (%)	34 (47.9)	8 (27.6)	26 (61.9)	0.004

Abbreviations: IV, intravenous.

**Table 3:** Neuroimaging and structural ophthalmologic findings at baseline.

Variable	Total (*n* = 71)	Favorable (*n* = 29)	Unfavorable (*n* = 42)	*p*-Value
Affected hemisphere				
Right, *n* (%)	32 (45.1)	13 (44.8)	19 (45.2)	0.972 (χ^2^)
Left, *n* (%)	32 (45.1)	14 (48.3)	18 (42.9)	—
Bilateral, *n* (%)	7 (9.9)	2 (6.9)	5 (11.9)	—
Lesion volume, mL	27.3 ± 17.6	18.7 ± 11.4	33.2 ± 18.7	<0.001
Optic radiation involvement, *n* (%)	31 (43.7)	9 (31.0)	22 (52.4)	0.073
Occipital lobe involvement, *n* (%)	34 (47.9)	18 (62.1)	16 (38.1)	0.046
Bilateral occipital, *n* (%)	5 (7.0)	1 (3.4)	4 (9.5)	0.396 (Fisher)
Baseline visual acuity (logMAR)	0.46 ± 0.21	0.39 ± 0.18	0.51 ± 0.22	0.018
Baseline visual field index, %	32.2 ± 14.7	38.4 ± 14.7	27.9 ± 13.1	0.003
Baseline Esterman score	41.7 ± 18.4	49.8 ± 17.6	36.1 ± 17.2	0.002
OCT pRNFL thickness, μm	82.7 ± 10.1	89.3 ± 7.6	78.2 ± 9.4	<0.001
OCT GCL thickness, μm	67.8 ± 8.4	72.4 ± 6.9	64.6 ± 8.2	<0.001

Abbreviations: GCL, ganglion cell layer; logMAR, logarithm of the minimum angle of resolution; OCT, optical coherence tomography; pRNFL, peripapillary retinal nerve fiber layer.

**Table 4:** Functional, visual, and quality-of-life outcomes at discharge and six-month follow-up.

Variable	Total (*n* = 71)	Favorable (*n* = 29)	Unfavorable (*n* = 42)	*p*-Value
NIHSS at discharge	5.3 ± 3.8	2.7 ± 1.9	7.1 ± 3.7	<0.001
mRS 0–2 at discharge, *n* (%)	31 (43.7)	21 (72.4)	10 (23.8)	<0.001
mRS 0–2 at 6 months, *n* (%)	35 (49.3)	22 (75.9)	13 (31.0)	<0.001
Visual field index at 6 months, %	51.4 ± 22.3	73.6 ± 11.2	36.1 ± 14.8	<0.001
Visual acuity at 6 months (logMAR)	0.31 ± 0.18	0.19 ± 0.11	0.39 ± 0.17	<0.001
ΔVFI (6 months − baseline), %	19.3 ± 18.6	35.2 ± 12.4	8.2 ± 11.7	<0.001
NEI VFQ-25 composite at 6 months	62.1 ± 18.4	78.3 ± 9.7	50.9 ± 14.6	<0.001
Time to visual rehab, days	17.5 ± 9.4	11.4 ± 5.3	21.7 ± 9.8	<0.001
Length of hospital stay, days	12.4 ± 6.3	9.7 ± 4.1	14.3 ± 6.7	0.001

Abbreviations: logMAR, logarithm of the minimum angle of resolution; mRS, modified Rankin Scale; NEI VFQ-25, National Eye Institute Visual Function Questionnaire-25; NIHSS, National Institutes of Health Stroke Scale; ΔVFI, change in visual field index.

**Table 5:** Univariable and multivariable logistic regression for predictors of favorable visual recovery at six months.

Variable (Scaling)	Univariable OR (95% CI)	*p*	Multivariable OR (95% CI)	*p*
Age (per 10 years)	0.43 (0.21–0.87)	0.018	0.62 (0.24–1.61)	0.327
NIHSS at admission (per 1 point)	0.71 (0.59–0.85)	<0.001	0.79 (0.62–0.99)	0.044
Hemorrhagic stroke (vs. ischemic)	0.32 (0.06–1.65)	0.173	—	—
PCA territory (vs. other)	4.01 (1.51–10.64)	0.005	2.92 (0.88–9.66)	0.078
Lesion volume (per 5 mL)	0.69 (0.56–0.85)	<0.001	0.81 (0.65–1.01)	0.064
Time to rehabilitation (per 7 days)	0.43 (0.27–0.69)	<0.001	0.52 (0.30–0.91)	0.022
Baseline VFI (per 10%)	1.79 (1.21–2.65)	0.004	1.42 (0.86–2.34)	0.169
OCT pRNFL (per 5 μm)	2.13 (1.42–3.19)	<0.001	1.81 (1.13–2.91)	0.014
OCT GCL (per 5 μm)	1.87 (1.31–2.67)	0.001	—	—

Model fit metrics: AIC = 67.2; Nagelkerke R^2^ = 0.583; Hosmer–Lemeshow *p* = 0.482. Abbreviations: CI, confidence interval; GCL, ganglion cell layer; NIHSS, National Institutes of Health Stroke Scale; OCT, optical coherence tomography; OR, odds ratio; PCA, posterior cerebral artery; pRNFL, peripapillary retinal nerve fiber layer; VFI, visual field index.

**Table 6:** Discrimination, calibration, and diagnostic performance of progressively richer predictive models.

Model	AUC (95% CI)	Sens (%)	Spec (%)	Acc (%)	PPV (%)	NPV (%)	Brier	HL *p*
Clinical only	0.71 (0.59–0.83)	65.5	71.4	69.0	61.3	75.0	0.213	0.371
Clinical + imaging	0.79 (0.69–0.89)	72.4	78.6	76.1	70.0	80.5	0.181	0.418
Clinical + imaging + ophthalmologic	0.84 (0.75–0.93)	79.3	81.0	80.3	74.2	85.0	0.158	0.547
Full integrated (+rehab/treatment)	0.87 (0.79–0.95)	82.8	85.7	84.5	80.0	87.8	0.142	0.624

HL *p*, Hosmer–Lemeshow goodness-of-fit *p*-value; PPV, positive predictive value; NPV, negative predictive value. Acc, accuracy; AUC, area under the receiver operating characteristic curve; CI, confidence interval; Sens, sensitivity; Spec, specificity.

**Table 7:** Vascular territory-stratified effects of key predictors with formal interaction testing.

Predictor/Territory	*n* (Favorable Rate)	OR per Unit (95% CI)	*p*	Interaction *p*
NIHSS per 1 point				
PCA territory	28 (60.7%)	0.66 (0.49–0.89)	0.007	0.041
MCA territory	23 (26.1%)	0.74 (0.58–0.95)	0.018	—
Vertebrobasilar	12 (25.0%)	0.88 (0.66–1.17)	0.379	—
Lesion volume per 5 mL				
PCA territory	28	0.62 (0.43–0.89)	0.010	0.067
MCA territory	23	0.71 (0.52–0.97)	0.034	—
Vertebrobasilar	12	0.84 (0.59–1.20)	0.341	—
Time to rehab per 7 days				
PCA territory	28	0.34 (0.16–0.72)	0.005	0.039
MCA territory	23	0.46 (0.21–1.01)	0.054	—
Vertebrobasilar	12	0.71 (0.34–1.48)	0.367	—
OCT pRNFL per 5 μm				
PCA territory	28	2.31 (1.36–3.92)	0.002	0.082
MCA territory	23	1.89 (1.07–3.34)	0.029	—
Vertebrobasilar	12	1.42 (0.81–2.49)	0.219	—

Abbreviations: CI, confidence interval; MCA, middle cerebral artery; NIHSS, National Institutes of Health Stroke Scale; OCT, optical coherence tomography; OR, odds ratio; PCA, posterior cerebral artery; pRNFL, peripapillary retinal nerve fiber layer.

**Table 8:** Cox proportional hazards regression for time to functional visual recovery (≥50% VFI improvement), with Kaplan–Meier estimates stratified by phenotype cluster.

Predictor		HR (95% CI)	*p*
Age (per 10 years)		0.71 (0.52–0.96)	0.027
NIHSS at admission (per 1 point)		0.83 (0.74–0.93)	0.001
Lesion volume (per 5 mL)		0.78 (0.66–0.92)	0.003
Hemorrhagic stroke (vs. ischemic)		0.34 (0.11–1.05)	0.061
Early rehabilitation ≤ 14 days (vs. >14)		2.41 (1.32–4.42)	0.004
OCT pRNFL ≥ 85 μm (vs. <85)		1.96 (1.12–3.43)	0.018
Baseline VFI (per 10%)		1.42 (1.04–1.93)	0.027
PCA territory (vs. other)		1.83 (1.04–3.22)	0.036
Kaplan–Meier estimates of median time to ≥50% VFI improvement by phenotype cluster:			
Cluster	*n*	Events/*n*	Median days (95% CI)
Cluster 1 (Localized-Posterior)	24	19/24	52 (43–71)
Cluster 2 (Multifocal/Mixed)	23	8/23	124 (87–NR)
Cluster 3 (Extensive/Neuroaxonal)	24	2/24	Not reached
Log-rank *p*			<0.001

Abbreviations: CI, confidence interval; HR, hazard ratio; NIHSS, National Institutes of Health Stroke Scale; NR, not reached; OCT, optical coherence tomography; PCA, posterior cerebral artery; pRNFL, peripapillary retinal nerve fiber layer; VFI, visual field index.

**Table 9:** Unsupervised k-means clustering identifies three reproducible post-stroke visual impairment phenotypes.

Variable	Cluster 1: Localized-Posterior (*n* = 24)	Cluster 2: Multifocal/Mixed (*n* = 23)	Cluster 3: Extensive/Neuroaxonal (*n* = 24)	*p*
Age, years	60.4 ± 9.7	67.8 ± 10.4	73.9 ± 10.2	<0.001
NIHSS at admission	5.3 ± 2.1	8.7 ± 3.4	12.6 ± 4.1	<0.001
Lesion volume, mL	13.8 ± 6.2	25.4 ± 9.7	42.1 ± 14.6	<0.001
OCT pRNFL, μm	91.7 ± 4.6	83.6 ± 6.3	75.4 ± 6.8	<0.001
Baseline VFI, %	43.2 ± 12.8	32.4 ± 11.6	21.8 ± 9.3	<0.001
PCA territory, *n* (%)	18 (75.0)	6 (26.1)	4 (16.7)	<0.001
MCA territory, *n* (%)	3 (12.5)	9 (39.1)	11 (45.8)	—
Hemianopia/quadrantanopia, *n* (%)	21 (87.5)	11 (47.8)	8 (33.3)	<0.001
Time to rehabilitation, days	11.7 ± 4.3	17.6 ± 7.8	23.4 ± 11.2	<0.001
Favorable recovery, *n* (%)	19 (79.2)	8 (34.8)	2 (8.3)	<0.001
VFI at 6 months, %	71.3 ± 13.2	48.7 ± 17.4	34.6 ± 13.7	<0.001
mRS 0–2 at 6 months, *n* (%)	19 (79.2)	12 (52.2)	4 (16.7)	<0.001
Mean predicted probability	0.79 ± 0.12	0.41 ± 0.16	0.16 ± 0.09	<0.001

Abbreviations: MCA, middle cerebral artery; mRS, modified Rankin Scale; NIHSS, National Institutes of Health Stroke Scale; OCT, optical coherence tomography; PCA, posterior cerebral artery; pRNFL, peripapillary retinal nerve fiber layer; VFI, visual field index.

## Data Availability

The data presented in this study are available upon reasonable request from the corresponding author.

## References

[1] Feigin V.L., Brainin M., Norrving B., Martins S., Sacco R.L., Hacke W., Fisher M., Pandian J., Lindsay P. (2022). World Stroke Organization (WSO): Global Stroke Fact Sheet 2022. Int. J. Stroke.

[2] (2021). GBD 2019 Stroke Collaborators. Global, regional, and national burden of stroke and its risk factors, 1990–2019: A systematic analysis for the Global Burden of Disease Study 2019. Lancet Neurol..

[3] Rowe F.J., Hepworth L.R., Howard C., Hanna K.L., Cheyne C.P., Currie J. (2019). High incidence and prevalence of visual problems after acute stroke: An epidemiology study with implications for service delivery. PLoS ONE.

[4] Sand K.M., Midelfart A., Thomassen L., Melms A., Wilhelm H., Hoff J.M. (2013). Visual impairment in stroke patients—A review. Acta Neurol. Scand..

[5] Pollock A., Hazelton C., Rowe F.J., Jonuscheit S., Kernohan A., Angilley J., Henderson C.A., Langhorne P., Campbell P. (2019). Interventions for visual field defects in people with stroke. Cochrane Database Syst. Rev..

[6] Hepworth L.R., Rowe F.J., Walker M.F., Rockliffe J., Noonan C., Howard C., Currie J. (2016). Post-stroke visual impairment: A systematic literature review of types and recovery of visual conditions. Ophthalmol. Res. Int. J..

[7] Jindahra P., Petrie A., Plant G.T. (2009). Retrograde trans-synaptic retinal ganglion cell loss identified by optical coherence tomography. Brain.

[8] Yamashita T., Miki A., Iguchi Y., Kimura K., Maeda F., Kiryu J. (2012). Reduced retinal ganglion cell complex thickness in patients with posterior cerebral artery infarction detected using spectral-domain optical coherence tomography. Jpn. J. Ophthalmol..

[9] Brandt T., Steinke W., Thie A., Pessin M.S., Caplan L.R. (2000). Posterior cerebral artery territory infarcts: Clinical features, infarct topography, causes and outcome. Multicenter results and a review of the literature. Cerebrovasc. Dis..

[10] Schiemanck S.K., Post M.W.M., Witkamp T.D., Kappelle L.J., Prevo A.J.H. (2005). Relationship between ischemic lesion volume and functional status in the 2nd week after middle cerebral artery stroke. Neurorehabilit. Neural Repair.

[11] Bernhardt J., Hayward K.S., Kwakkel G., Ward N.S., Wolf S.L., Borschmann K., Krakauer J.W., Boyd L.A., Carmichael S.T., Corbett D. (2017). Agreed definitions and a shared vision for new standards in stroke recovery research: The Stroke Recovery and Rehabilitation Roundtable taskforce. Int. J. Stroke.

[12] (2013). Stroke Unit Trialists’ Collaboration. Organised inpatient (stroke unit) care for stroke. Cochrane Database Syst. Rev..

[13] Adams H.P., Davis P.H., Leira E.C., Chang K.-C., Bendixen B.H., Clarke W.R., Woolson R.F., Hansen M.D. (1999). Baseline NIH Stroke Scale score strongly predicts outcome after stroke: A report of the Trial of Org 10172 in Acute Stroke Treatment (TOAST). Neurology.

[14] Banks J.L., Marotta C.A. (2007). Outcomes validity and reliability of the modified Rankin Scale: Implications for stroke clinical trials—A literature review and synthesis. Stroke.

[15] Zhang X., Kedar S., Lynn M.J., Newman N.J., Biousse V. (2006). Natural history of homonymous hemianopia. Neurology.

[16] Rowe F.J., Conroy E.J., Bedson E., Cwiklinski E., Drummond A., García-Fiñana M., Howard C., Pollock A., Shipman T., Dodridge C. (2017). A pilot randomized controlled trial comparing effectiveness of prism glasses, visual search training and standard care in hemianopia. Acta Neurol. Scand..

[17] Sabel B.A., Henrich-Noack P., Fedorov A., Gall C. (2011). Vision restoration after brain and retina damage: The “residual vision activation theory”. Prog. Brain Res..

[18] Goodwin D. (2014). Homonymous hemianopia: Challenges and solutions. Clin. Ophthalmol..

[19] Saionz E.L., Tadin D., Melnick M.D., Huxlin K.R. (2020). Functional preservation and enhanced capacity for visual restoration in subacute occipital stroke. Brain.

[20] Mitchell A.G., Rossit S., Pal S., Hornberger M., Warman A., Kenning E., Williamson L., Shapland R., McIntosh R.D. (2020). Peripheral visual field loss after occipital stroke: The role of compensatory eye movements and visuospatial cognition. Neuropsychologia.

[21] Park J.H., Park S.W., Kang S.H., Nam T.K., Min B.K., Hwang S.N. (2018). Detection of trans-synaptic retrograde degeneration of retinal ganglion cells in patients with occipital infarction. Neuroophthalmology.

[22] Stroke Foundation of Europe, SAFE (2021). Stroke services in Europe: Comparison of stroke care quality across countries—The ESO-EAST programme. Eur. Stroke J..

[23] Caplan L.R., Wityk R.J., Glass T.A., Tapia J., Pazdera L., Chang H.-M., Teal P., Dashe J.F., Chaves C.J., Breen J.C. (2004). New England Medical Center Posterior Circulation Registry. Ann. Neurol..

[24] Howard C., Rowe F.J. (2018). Adaptation to poststroke visual field loss: A systematic review. Brain Behav..

[25] Yiu E.M., Hocking D.R., Storey E., Williamson R., Petty S.J. (2022). Trans-synaptic retrograde degeneration in post-stroke homonymous hemianopia: A longitudinal optical coherence tomography study. J. Neurol..

[26] Stinear C.M., Smith M.-C., Byblow W.D. (2019). Prediction tools for stroke rehabilitation. Stroke.

[27] Boyd L.A., Hayward K.S., Ward N.S., Stinear C.M., Rosso C., Fisher R.J., Carter A.R., Leff A.P., Copland D.A., Carey L.M. (2017). Biomarkers of stroke recovery: Consensus-based core recommendations from the Stroke Recovery and Rehabilitation Roundtable. Int. J. Stroke.

[28] Vickers A.J., Van Calster B., Steyerberg E.W. (2016). Net benefit approaches to the evaluation of prediction models, molecular markers, and diagnostic tests. BMJ.

[29] Collins G.S., Reitsma J.B., Altman D.G., Moons K.G.M. (2015). Transparent reporting of a multivariable prediction model for individual prognosis or diagnosis (TRIPOD): The TRIPOD statement. Ann. Intern. Med..

[30] Steyerberg E.W., Harrell F.E. (2016). Prediction models need appropriate internal, internal–external, and external validation. J. Clin. Epidemiol..

